# Supplementary material for: Does environmental information disclosure promote enterprise green technology innovation?
Source: PLoS One. 2024 Dec 13;19(12):e0312901. doi: 10.1371/journal.pone.0312901 (PMC11642913; doi:10.1371/journal.pone.0312901)
Supplement: Attachment — Submitted filename: Response to Reviewers.docx [file pone.0312901.s001.docx]

**Dear Editor and Reviewers,**

We are very grateful for the valuable feedback and insightful comments provided on our manuscript. We appreciate the time and effort you have invested in reviewing our work.

We have carefully considered all the comments and suggestions and have made substantial revisions to the manuscript accordingly. Below, we provide detailed responses to each point raised.

Thank you for your consideration.

**Editor Comments:**

1. Comment: "Manuscript is well written. However, Author must incorporate following comments along with reviewer suggestions: 1. English proof read required 2. Introduction should be revised and address research gap deeply. 3. Discuss methodology section more focused and explain variable. 4. Reference should be updated."

Response:

1. We have thoroughly proofread the revised manuscript in English and corrected grammatical errors.

2. In the introduction section, we have added relevant studies on green technology innovation to further highlight the importance of the research problem addressed in this paper.

3. We have expanded and optimized the methodology section, providing a more detailed explanation of the explanatory variables.

4. We have reviewed and updated the references to include the most recent and relevant literature.

**Reviewer 1 Comments:**

1. Comment: "The abstract should be in structured form and contain the main crux of the study."

Response: We appreciate this comment and have restructured the abstract to clearly define the research question and its significance.

2. Comment: "The author has not provided an adequate description of the potential effects of green technology on various sectors or industries."

Response: We have expanded the introduction to include information on the impact of green technology innovation across various sectors, emphasizing its importance and necessity.

3. Comment: "The author has employed excessive line spacing, leading to an increased page count. It is advisable to eliminate the additional line spacing to optimize the document's formatting and page count."

Response: We have revised the document to single-spacing, addressing the issue of excessive line spacing.

4. Comment: "The study does not provide future directions which are essential for any research study."

Response: Considering the study's limitations, we have included guidance for future research directions in Section 5.

5. Comment: "Please make the concluding section shorter and a little bit longer."

Response: We have expanded the conclusion section to provide a more comprehensive summary of our findings.

6. Comment: "Please compare the findings with those of other studies."

Response: In the discussion section of Section 5, we have compared our findings with previous research, highlighting the uniqueness and contributions of our study.

7. Comment: "In the final manuscript, a majority of the Figures and Tables are not cited in the main text. Please ensure that these are properly cited in the DOC format under the Main Manuscript section on Phenom to align with best practices."

Response: Figures have been placed within the main text of the manuscript and properly cited.

8. Comment: "On what basis were the companies from the China Stock Market selected? Please provide the complete source of data collection."

Response: We have outlined the criteria for selecting Chinese stock market firms and explained the basis for the study's timeframe.

9. Comment: "References should be alphabetically ordered for clarity and alignment with journal guidelines."

Response: The format and arrangement of references have been revised according to PLOS ONE journal guidelines.

10. Comment: "The author has overlooked including DOI numbers for several references. Please ensure that DOI numbers are added to enhance the comprehensiveness of the citation information."

Response: DOI numbers have been added to all references in the revised manuscript.

11. Comment: "Please make the appropriate adjustments to the references' in-text citation styles."

Response: The citation format of references has been standardized.

12. Comment: "The author should update the manuscript with more recent references and include the following additional references."

Response: We have updated the references. Based on the reviewers' suggestions, we have cited the following documents: doi:10.1155/2023/9536571 and doi:10.3389/fpsyg.2022.953454. Other references have also been updated in the revised manuscript.

13. Comment: "All of the references in the final list should be written in the same style."

Response: All references in the final list have been written in the same style.

14. Comment: "The paper's presentation might need some improvement. Please correct the writing and spacing errors."

Response: Writing and spacing errors have been corrected.

15. Comment: "The paper contains several grammatical errors. Please fix these, especially in the Introduction, Literature Review, and Conclusion."

Response: Grammatical errors have been corrected in the Introduction, Literature Review, and Conclusion sections.

16. Comment: "Please include in-text citations for all the tables and figures correctly and upload the final updated manuscript."

Response: All tables and figures have been correctly cited in the final manuscript.

17. Comment: "Provide clearer pictures about all Figures. Further, need to explain the meaning of the existence of figures."

Response: We provided clear pictures of all the figures and explained their significance in the context of the content.

18. Comment: "The discussion part of the research is missing. Make a heading only 'Discussion' and then write the discussion align with your study results. Authors need to search the latest literature and write the discussion section."

Response: We have added a discussion section to analyze relevant studies by other scholars and to highlight the significance of this study.

**Reviewer 2 Comments:**

1. Comment: "Thank you for inviting me to review this work. I thoroughly reviewed this work and found it interesting and meaningful with merits. This study provides valuable insights for countries with imperfect environmental governance systems. Based on study design, model, and analysis, I would recommend it for publication with minor language editing."

Response: We are grateful for your positive feedback and recommendation. We have made minor language edits throughout the manuscript to improve clarity and readability.

**Summary of changes:**

1. Revised the abstract to clarify the research question, significance and main conclusions.

2. Expanded the introduction section to include the economic and environmental roles brought about by green technology innovations, and to more clearly state the research question of this paper (p. 1, para. Page 2, paragraph 1).

3. The methodology section was revised to explain the data selection and the starting and ending points of the study. Also added details on the selection of explanatory variables. (Page 6, para. Page 8, paragraphs 3-4)

4. Expanded the conclusion section. A new discussion department was added to clarify the significance of the research in this paper by combining the existing studies and the conclusions of this paper. In the end, an outlook for future research based on the limitations of this paper was added. (Page 18, para. 1-2. Page 20, paragraph 1)

During the revision writing process, we received help from other scholars. Therefore, we have adjusted the author's information. All authors agreed upon this resolution.

In summary, we believe these revisions have significantly improved the manuscript. Thank you again for your constructive feedback. We look forward to your further comments.

Best regards,

Xingqi Wang

Xingqiwang1030@163.com
