# Supplementary material for: Does environmental information disclosure promote enterprise green technology innovation?
Source: PLoS One. 2024 Dec 13;19(12):e0312901. doi: 10.1371/journal.pone.0312901 (PMC11642913; doi:10.1371/journal.pone.0312901)
Supplement: Attachment — Submitted filename: Response to Reviewers.docx [file pone.0312901.s002.docx]

**Response to Reviewers**

**Dear Editor and Reviewers,**

Firstly, thank you for the time and effort spent reviewing our manuscript. Your insightful comments and detailed feedback have been crucial in improving our work. We have carefully considered your suggestions and made the necessary revisions. We hope these changes meet your expectations and aid in the manuscript’s publication.

In this revision, we particularly emphasized the direct and indirect impacts of Environmental Information Disclosure (EID) on green technology innovation, explicitly revealing the mechanisms through which it operates alongside other environmental regulatory factors. Additionally, we have meticulously optimized the logical expression to ensure clarity and coherence in our discussion. Linguistically, we refined our wording based on the reviewers’ suggestions and conducted a thorough review and update of the reference formatting to meet academic standards.

To clearly demonstrate these modifications, we employed revision markings to visually indicate all the changes made. We hope these efforts meet the reviewers’ expectations and contribute to the acceptance and publication of our paper.

Next, we elaborate on the changes based on the reviewers’ suggestions

**Reviewer #1**:

Author incorporate all the changes highlighted in comments. The author incorporate the changes and fix these in effective manner. Now the time to publish the author work I highly recommend it.

**Response:** Thank you for your positive feedback and for endorsing the publication of our work. We are grateful for your thorough review and guidance throughout the revision process.

**Reviewer #3**:

1.The innovation point of this article needs to be more precise. There is already some clear literature on the correlation between PITI and green technology innovation. The author needs to seek their unique features humbly.

**Response:** We revised the innovation points. The innovations were further summarized. Also only the value of the existing research was outlined. The specific modifications are as follows:

This study offers several key contributions. First, a multi-period DID model is employed to assess the overall impact of EID on enterprise green technology innovation. Previous studies have explored the relationship between EID and green innovation, often using single-period DID models [28]. This study, however, accounts for the effects of new market entrants and the relocation of existing firms, addressing a gap in prior research. Second, this study examines how EID influences the implementation of other environmental regulatory tools. While previous research has analyzed the impact of EID on technology innovation through mechanisms such as human capital, foreign direct investment [30], political pressures, enforcement channels [29], innovation environment, investment, talent [31], green innovation environment, industrial structure [16], and corporate social responsibility [28], the interaction between different regulatory tools remains underexplored. To fill this gap, the study investigates how EID enhances public participation-based, command-control, and market-incentive regulations, analyzing whether EID can strengthen other regulatory tools to promote green technology innovation.

2.For the multi period DID model, the construction of Model 1 in this article is incorrect and requires significant adjustments. As this article uses multi period DID, it’s crucial to provide rigorous evidence based on the current research progress of methods. The current recognition results are not reliable because only the case of classical DID is considered.

**Response:**We used a multi-temporal DID model, and we optimized the model and formulation based on the reviewers’ comments.

After modification:

 (1)

 (2)

 (3)

Before modification:

$GI_{it}=\beta_{0}+\beta_{1}Treat_{i}\times Period_{t}+\beta_{2}Control_{it}+\gamma_{i}+\delta_{t}+\mu_{it}$ (1)

$M_{it}=\theta_{0}+\theta_{1}Treat_{i}\times Poriod_{t}+\theta_{2}Control_{it}+\gamma_{i}+\delta_{t}+\mu_{it}$ (2)

 (3)

3.Why does local governments’ disclosure of environmental information directly lead to green technology innovation in enterprises? Whether in the theoretical hypothesis discussion in this article or in the empirical strategies later, this theory and logic have not provided satisfactory answers. According to relevant literature, the vast majority of enterprises are completely unaware of this PITI. If this article believes that PITI can affect the quality of green innovation, what is the specific path?

**Response:** We refined our original logic. Rewrote the hypothesis part of our research. And we have rewritten the logic of PITI affecting the green technology innovation of enterprises, summarizing in addition to the influential role of PITI. And, we rewrote the content of mechanism analysis. The specific modifications are as follows:

The existing research on the impact of environmental regulation on enterprise innovation forms the theoretical basis of this study. Neoclassical economic theory suggests that environmental regulations increase enterprises’ “compliance costs,” potentially hindering green innovation. In contrast, the “Porter Hypothesis” argues that well-designed regulations can encourage green technology innovation through an “innovation compensation” effect. Therefore, this study hypothesizes that EID positively influences enterprise green technology innovation.

Firstly, EID promotes enterprise green technology innovation through legitimacy pressures and reputation mechanisms. According to legitimacy theory, enterprises must follow social norms and environmental regulations to maintain their market legitimacy and reputation [42]. EID increases transparency by disclosing information about pollution and violations, subjecting enterprises to public and investor scrutiny. If the enterprise’s actions deviate from expectations, its legitimacy and market position may suffer, leading to potential losses in market share and investor trust [16]. As a result, enterprises need to adopt green technology that goes beyond regulatory requirements to build a responsible image [43].

Secondly, EID compels firms to engage in green technology innovation by internalizing externalities and maximizing long-term returns. Based on cost-benefit analysis theory, firms weigh investment costs against the direct and indirect benefits of innovation when making decisions regarding green technology investments. The transparency mechanism of EID directly transfers the costs of environmental pollution to enterprises, compelling them to assume greater environmental responsibilities [28]. In this context, enterprises consider the future costs of polluting behaviors and reassess the long-term benefits of green technology innovation. This reevaluation amplifies the perceived long-term gains from green technology innovation, encouraging firms to undertake green technology innovation activities.

Lastly, EID fosters industry-wide green technology advancement through competitive effects. By making environmental information transparent, EID shifts the focus of competition within industries from traditional factors like price and quality to environmental performance [44, 45]. Leading enterprises gain a “first-mover advantage” through green technology innovation, while other firms follow suit or pursue further innovation. This creates a demonstration and competitive effect within the industry, driving the overall upgrade and transition towards green technology.

4.In 3.3.3 Mechanism variable, both government-led environmental regulation and public participation-based environmental regulation are considered instrumental variables, which conflicts with the title.

**Response:** We are very sorry that we had a problem with the text, government-led environmental regulation and public participation-based environmental regulation are actually mechanism variables. And, we refine government-led environmental regulation into command-control and market-incentive environmental regulation. Thanks to the reviewer’s care, we have made the correction.

5.What is the basis for selecting public participation-based environmental regulation and government-led environmental regulation in the 4.4 Mechanism Analysis section, and which category should the PITI in this article belong to? We should not be enthusiastic about creating concepts but focus on rigorous argumentation.

**Response:** We are very sorry that our selection of mechanism variables appeared unsupported in the previous version. Now, we have supplemented it and added some literature to support the basis of the selection in this paper. We summarize the existing classification of environmental regulatory tools. According to the different implementation subjects of environmental regulatory tools, environmental regulatory tools can be categorized into two types: government-led environmental regulation, i.e., command-control environmental regulation, and market-incentive environmental regulation. The second is informal environmental regulation with non-government participation, including public participation-based environmental regulation, voluntary environmental regulation, and information disclosure mechanisms. This paper mainly explores the impact of environmental regulatory tools on green technology innovation from the outside of enterprises, so the voluntary environmental regulation of enterprises is outside the scope of this paper. In this paper, we focus on assessing the EID of cities through the Pollution Source Monitoring Information Disclosure Index (PITI). This type of environmental regulation belongs to the information disclosure mechanism.

The specific choices of command-control environmental regulation, market-incentive environmental regulation, and public participation-based environmental regulation are based on the following:

The first mechanism variable is public participation-based environmental regulation. Public participation-based environmental regulation emphasizes the voluntary actions of market participants in controlling pollution. Existing studies typically use the number of public complaints and environmental proposals related to environmental issues as measurement indicators [16,24,57]. Therefore, this study uses the natural logarithm of the number of environmental proposals in the city where the firm is located as a proxy for public participation-based environmental regulation.

The second mechanism variable is command-control environmental regulation. Command-control environmental regulation refers to government-imposed laws and environmental standards that limit corporate pollutant emissions to promote environmental protection. Prior literature commonly uses the number of environmental administrative penalties to measure the stringency of this regulation [48,57]. Similarly, this study uses the natural logarithm of the number of environmental administrative penalties (plus one) in the city where the firm is located to capture the intensity of command-control regulation.

The third mechanism variable is market-incentive environmental regulation. Market-incentive environmental regulation leverages market pricing mechanisms to incentivize firms to assume environmental responsibility and consider pollution control in their production processes. Scholars frequently use the amount of pollution fees as a key indicator of this regulation [24,58,59]. Following this approach, we employ the natural logarithm of the pollution fees in the city where the firm is located to measure the strength of market-incentive environmental regulation.

6.The citation of references in this article is inaccurate, and the language expression requires professional polishing to make the article more readable.

**Response:** We checked the formatting of the references through Google Scholar and made corrections for some grammar and wording.

Once again, thank you for your valuable feedback and guidance. We are grateful for the critiques and directions provided, which have significantly helped to deepen our research and refine our manuscript. We look forward to your assessment of these revisions and are more than willing to make further modifications to meet the journal’s standards for publication. Please do not hesitate to provide additional guidance.

Sincerely,

Xingqi Wang

School of Economics and Management

Shihezi University

xingqiwang1030@163.com

October 12, 2024
